# Supplementary material for: Carboxamide Derivatives Are Potential Therapeutic AHR Ligands for Restoring IL-4 Mediated Repression of Epidermal Differentiation Proteins
Source: Int J Mol Sci. 2022 Feb 4;23(3):1773. doi: 10.3390/ijms23031773 (PMC8836151; doi:10.3390/ijms23031773)
Supplement: Supplementary file 1 [file ijms-23-01773-s001.zip › ijms-1526583-supplementary.pdf]

Supplemental Table S1. Overview of quinoline-3-carboxamide derivatives for the study on epidermal differentiation induction. The structural relationship between the parent compound (LAQ, TASQ or ROQ) and its newly developed derivatives is depicted with color codes and in the comments column.

Compound Abbreviation	Chemical Structure	Scientific Name	Comments
Laquinimod (LAQ)	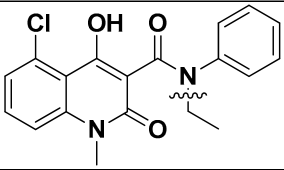	5-chloro-*N*-ethyl-4-hydroxy-1-methyl-2-oxo-*N*-phenyl-1,2-dihydroquinoline-3-carboxamide	Site of N-dealkylation indicated in structure
IMA-06201	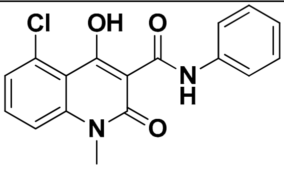	*N*-phenyl-5-chloro-1,2-dihydro-4-hydroxy-1-methyl-2-oxo-quinoline-3-carboxamide	N-dealkylated metabolite of LAQ (DELAQ in lit.). Intramolecular H-bonds stabilizes a planar structure
IMA-08401	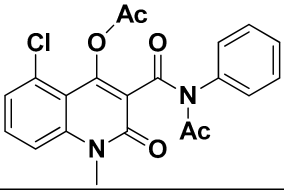	*N*-acetyl-*N*-phenyl-4-acetoxy-5-chloro-1,2-dihydro-1-methyl-2-oxo-quinoline-3-carboxamide	Di-acetyl prodrug of IMA-06201
Tasquinimod (TASQ)	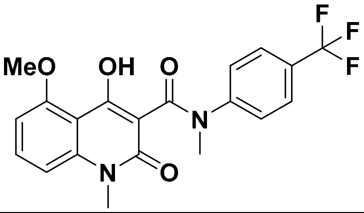	4-hydroxy-5-methoxy-*N*,1-dimethyl-2-oxo-*N*-[4-(trifluoromethyl)phenyl]quinoline-3-carboxamide	Aimed for the treatment of prostate cancer
IMA-06504	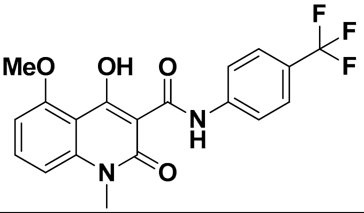	*N*-(4-trifluoromethylphenyl)-1,2-dihydro-4-hydroxy-5-methoxy-1-methyl-2-oxo-quinoline-3-carboxamide	N-dealkylated metabolite of TASQ
IMA-07101	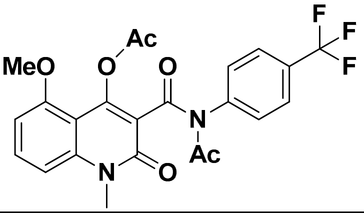	*N*-acetyl-*N*-(4-trifluoromethylphenyl)-4-acetoxy-1,2-dihydro-5-methoxy-1-methyl-2-oxo-quinoline-3-carboxamide	Di-acetyl prodrug of IMA-06504
Roquinimex (ROQ)	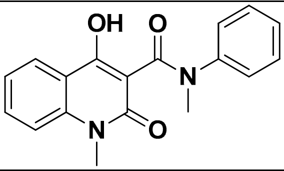	4-hydroxy-*N*,1-dimethyl-2-oxo-*N*-phenylquinoline-3-carboxamide	First clinical compound (Linomide) in class
IMA-05101	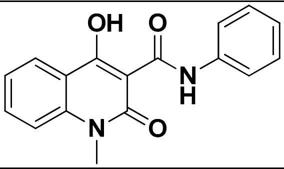	*N*-phenyl-1,2-dihydro-4-hydroxy-1-methyl-2-oxo-quinoline-3-carboxamide	N-dealkylated metabolite of ROQ

IMA-01403	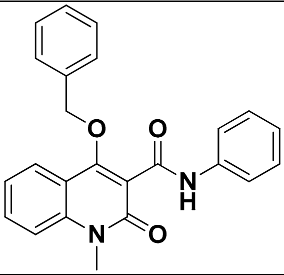	*N*-phenyl-4-benzyloxy-1,2-dihydro-1-methyl-2-oxo-quinoline-3-carboxamide	Low-potency ligand. 4-O-benzyl breaks H-bond
TCDD	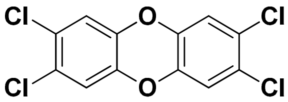	2,3,7,8-tetrachlorodibenzo-p-dioxin	The metabolically resistant compound "dioxin"

Supplemental Table S2. Human primary keratinocytes used to generate the AD-HEEs harboring a heterozygous *FLG* mutation leading to depicted amino acid change.

**Sample ID**	***FLG* Mutation**	**Amino acid Change**
Donor#1	c.7339C>T	p.(Arg2447*)
Donor#2	c.5702del	p.(Gly1901Alafs*194)
Donor#3	c.10898C>G	p.(Ser3633*)

Supplemental Table S3. Antibodies used of immunohistochemical analysis.

Antigen	Species	Dilution	Company
**Filaggrin**	Mouse	1:100	Thermo Fisher, MA5-13440
**CYP1A1**	Mouse	1:50	Santa Cruz, sc-25304
**Involucrin**	Mouse	1:20	Van Duijn hoven
**Loricrin**	Rabbit	1:4000	Convance 145P100

Supplemental Table S4. Primers used of RT-qPCR analysis.

Gene	Forward Primer (5'-3')	Reverse Primer (5'-3')
HARP	caccattgaaatcctgagtgatgt	tgaccagcccaaaggagaag
CYP1A1	ctggagaccttccgacactctt	gtaaaagcctttcaaacttgtgtctct
FLG	acttcactgagtttcttctgatggtatt	tccagacttgagggtctttttctg
HRNR	tgttcctctggtgagctaggttact	tgggtggcatattggtagaaaac
IVL	acttatttcgggtccgctaggt	gagacatgtagagggacagagtcaag
LOR	aggttaagacatgaaggatttgcaa	ggcaccgatgggcttagag

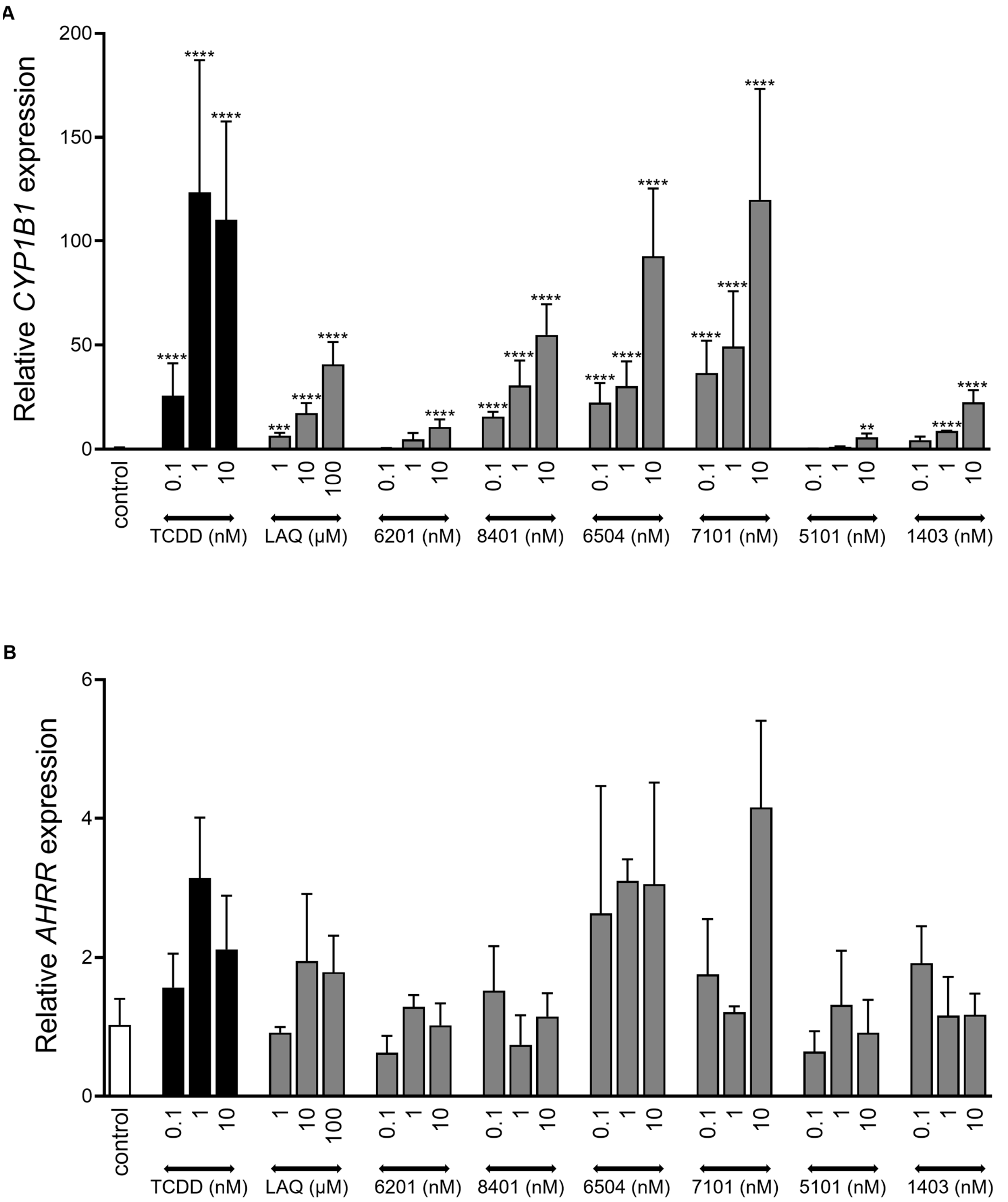

Supplemental Figure S1. Analysis of AHR target genes. mRNA expression analysis of **(A)** *CYP1B1* and **(B)** *aryl hydrocarbon receptor repressor* (*AHRR*) after 48 hours stimulation (re-stimulation after 24 hours) of monolayer primary human keratinocytes (N=3) with a concentration series of the IMA-compounds, LAQ and TCDD. *P<0.05, **P<0.01, ***P<0.001, ****P<0.0001. Mean +/- SEM.

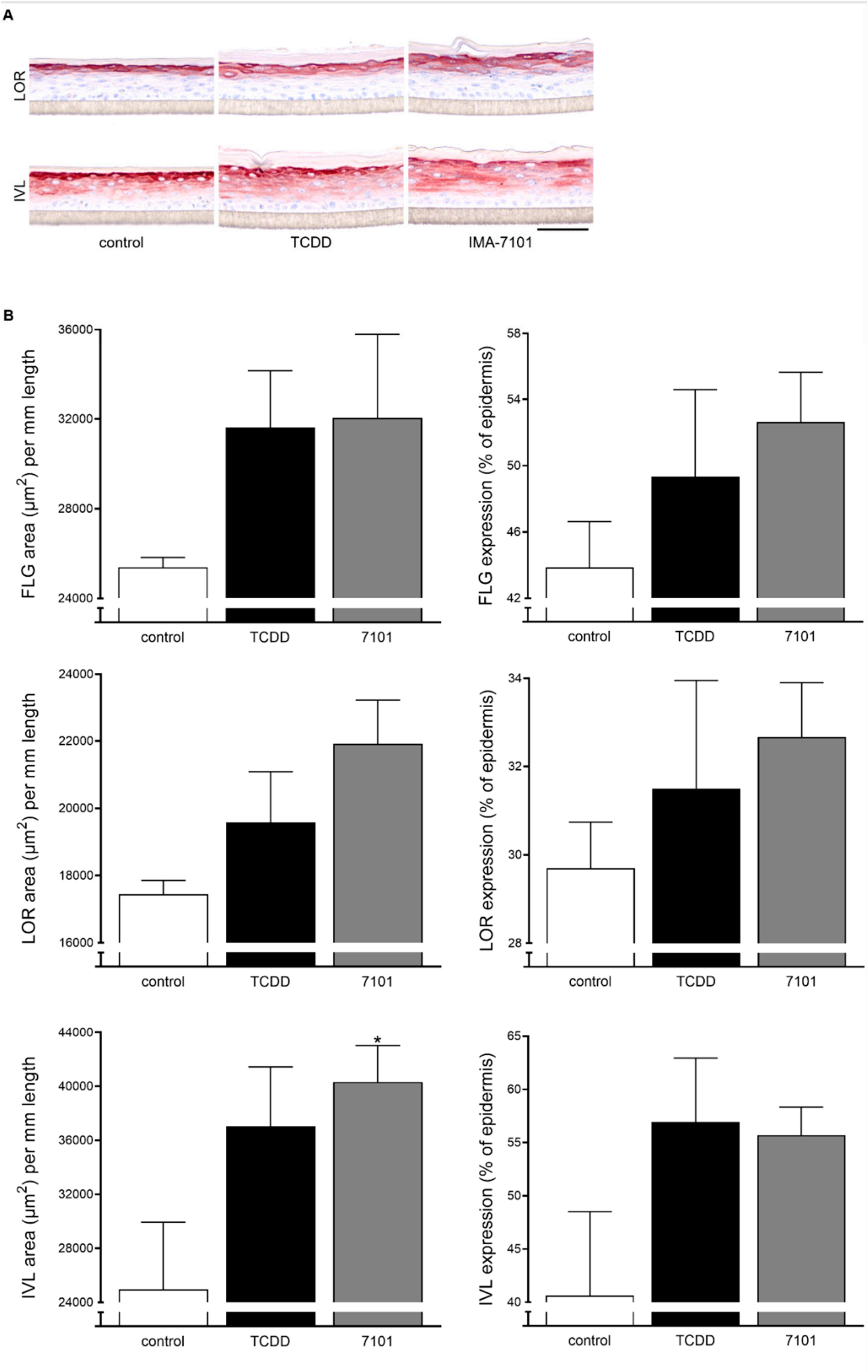

Supplemental Figure S2. Epidermal differentiation analysis in HEE model. **(A)** loricrin (LOR) and involucrin (IVL) staining of HEEs stimulated with 1 nM TCDD and IMA-07101 for 96 hours and **(B)** quantification thereof (µm² per mm length of the epidermis and percentage expression of the epidermis), including filaggrin (FLG) expression (N=3). *P<0.05. Mean +/- SEM. Scale bar = 100 µm.

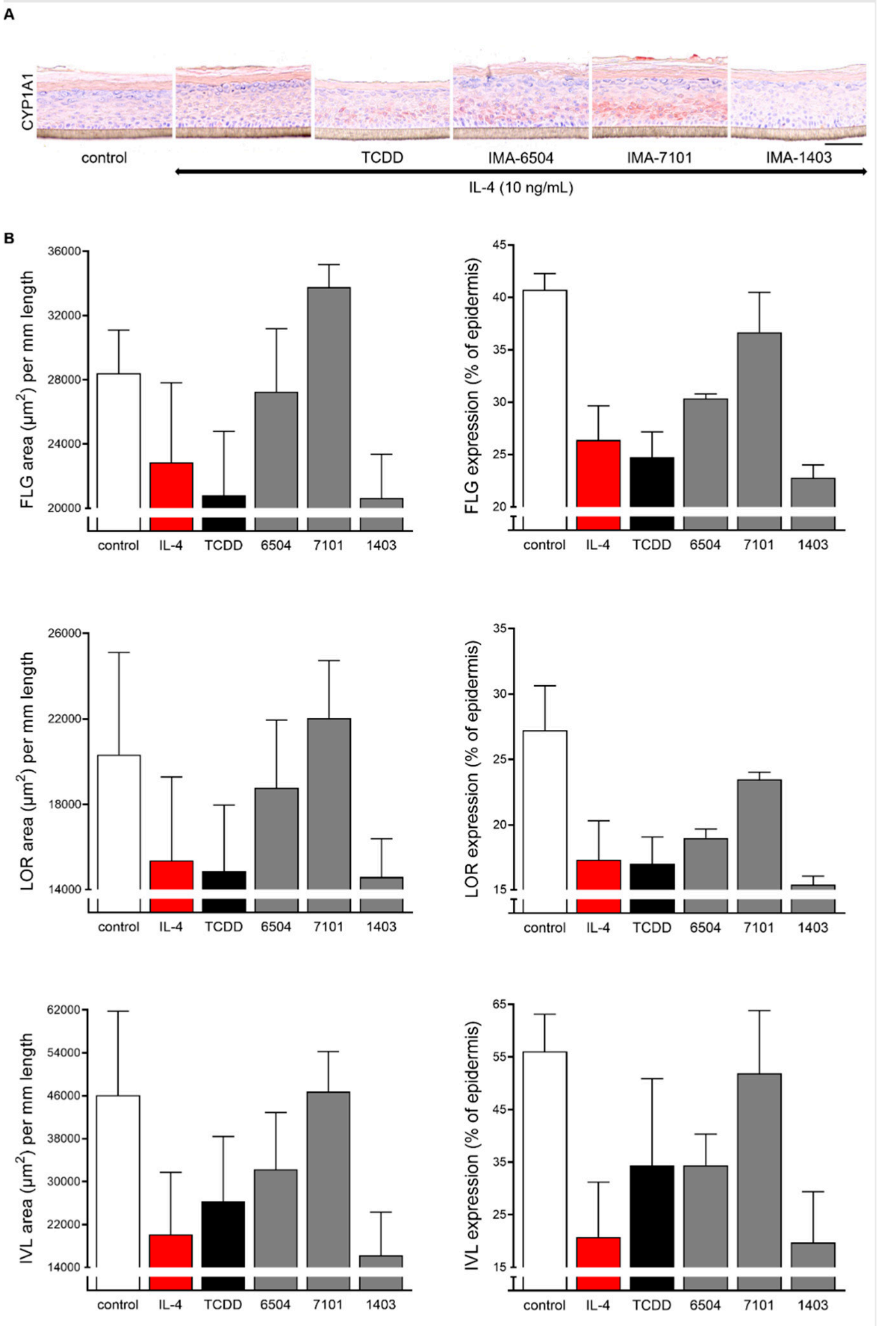

Supplemental Figure S3. AHR-mediated restoration of differentiation protein expression. **(A)** immunostaining for CYP1A1 and **(B)** quantification (μm[2] per mm length of the epidermis and percentage expression of the epidermis) of protein expression for filaggrin (FLG; P = 0.032, N=3)), Loricrin (LOR) and Involucrin (IVL; P = 0.025) in HEEs stimulated with 10 ng/mL IL-4,for 24 hours followed by co-stimulation with the compounds for another 72 hours. Scale bar = 100 μm.
